# Numerical Simulation and Borehole Azimuthal Acoustic Imaging of Near-Borehole Caves in Formations with Axially Non-Uniform Wave Velocities

**DOI:** 10.3390/s26144637

**Published:** 2026-07-22

**Authors:** Bo Yang, Xiaohua Che, Teng Zhao, Junqiang Lu, Baiyong Men, Wenxiao Qiao

**Affiliations:** 1 State Key Laboratory of Petroleum Resources and Engineering, China University of Petroleum (Beijing), Beijing 102249, China; ygbocup@126.com (B.Y.); zhaotengchn@foxmail.com (T.Z.); lujq@cup.edu.cn (J.L.); bymen@cup.edu.cn (B.M.); qiaowx@cup.edu.cn (W.Q.); 2 Key Laboratory of Earth Prospecting and Information Technology, Beijing 102249, China

**Keywords:** acoustic logging, numerical simulation, ray tracing, spatial scanning

## Abstract

During the exploration and development of oil and gas fields, near-borehole formations exhibit considerable axial heterogeneity in elastic-wave velocity. However, existing borehole azimuthal acoustic imaging methods often ignore the effect of this heterogeneity on imaging performance and thus cannot accurately locate anomalous near-borehole bodies in formations with axially non-uniform wave velocities. Hence, a borehole azimuthal acoustic imaging method based on ray tracing and spatial scanning was developed to resolve this problem. Subsequently, the borehole azimuthal acoustic imaging responses of near-borehole caves in formations with axially uniform and axially non-uniform wave velocities were numerically simulated. Single-shot spatial-scanning imaging and multi-shot stack imaging were then implemented separately for PP (incident P-waves scattered as P-waves) scattered-echo data obtained from forward modelling. The results revealed that in formations with axially uniform velocities, the waveforms of scattered echoes from the near-borehole caves exhibited a typical parabolic variation as a function of depth. Conversely, in formations with axially non-uniform velocities, the waveforms exhibited an asymmetric, curved variation pattern with respect to depth. The conventional downhole three-dimensional spatial-scanning method could not accurately locate the near-borehole anomalies in formations with axially non-uniform wave velocities, producing large imaging errors for the simulated caves. By contrast, the proposed imaging method more precisely determined the radial distances, azimuths and depths of the simulated caves. The proposed method may broaden the application scope of acoustic remote detection logging in the exploration and development of complex heterogeneous reservoirs.

## 1. Introduction

Acoustic remote detection logging identifies small geological structures, such as fractures, caves and formation interfaces, located several to tens of metres away from boreholes, by analysing reflected and scattered echoes received using multi-channel arrays. Because its detection resolution and radial depth fall between those of conventional logging and seismic exploration systems, this technology bridges the technological gap between the exploration and development of oil and gas fields; consequently, it has become an indispensable advanced technique for formation evaluation in the industry [1,2].

Depending on the structure of the acoustic system, acoustic remote detection logging can be categorised into monopole P-wave remote detection, dipole S-wave remote detection and borehole azimuthal acoustic imaging. Monopole P-wave remote detection can only determine the distance from an anomalous body to the borehole and provides no azimuthal information because its source and receivers operate in a fully omnidirectional monopole vibrational mode [3]. Traditional dipole S-wave remote detection determines the development depth, dip angle and trend of anomalous near-borehole bodies using an acoustic system with orthogonal dipole transmission and reception capabilities; however, its azimuth measurement signal of near-borehole anomalous bodies features an inherent 180° ambiguity [4,5,6,7,8]. By contrast, borehole azimuthal acoustic imaging is used for the three-dimensional (3D) imaging of near-borehole formations because it measures the distance from the anomalous body to the borehole and the azimuthal angle while recording echo waveforms from different directions and evaluating near-borehole formations in the axial, circumferential and radial directions [9,10,11].

The current borehole azimuthal acoustic imaging methods and associated data-processing techniques include acoustic remote detection migration imaging, downhole 3D acoustic-wave directional scanning–receiving (beamforming), 3D far-field acoustic-wave imaging and 3D spatial-scanning imaging. However, most of these methods assume that a near-borehole formation is homogeneous and neglect the strong axial heterogeneity of elastic-wave velocity around the borehole [12,13,14,15,16]. This simplification is problematic in practice because boreholes often pass through multiple formations with different elastic-wave velocities. Consequently, reflected or scattered echoes from an anomalous near-borehole body undergo multiple refraction, reflection and scattering events at formation interfaces before reaching receivers and therefore no longer behave as plane waves. Thus, axial heterogeneity in elastic-wave velocity in near-borehole formations compromises the ability of traditional methods of borehole azimuthal acoustic imaging to accurately localise such bodies. Additionally, migration results contain false-reflection artefacts caused by formation interfaces, which affect imaging and inversion analysis outcomes [17]. Although the propagation of borehole mode waves in structures with axially non-uniform wave velocities has been extensively investigated worldwide [18,19,20,21], only a few reports have described borehole azimuthal acoustic imaging in formations with axially non-uniform wave velocities [22,23].

To overcome the limitations of traditional methods for borehole azimuthal acoustic imaging, this article proposes a borehole azimuthal acoustic imaging method for formations with axially non-uniform wave velocities. Numerically simulated waveform data are used for inversion and imaging. The advantages of the proposed method are evaluated by comparing its results with those of conventional 3D spatial-scanning imaging.

## 2. Methods

### 2.1. Ray Tracing of Echoes for Identifying Near-Borehole Scattering Points

**Figure 1:**
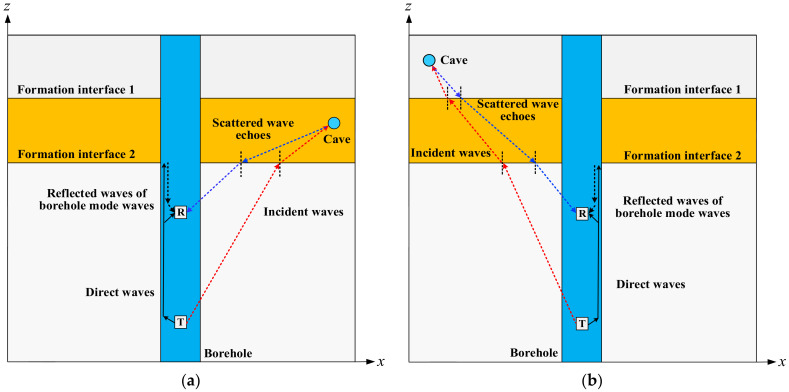
Propagation paths of incident waves and scattered echoes in a double-interface formation with axially non-uniform wave velocity: cave (**a**) within and (**b**) outside the interlayer.

When a pulsed acoustic wave generated by an acoustic source (T) in a liquid-filled borehole occurs on a near-borehole formation with an axially non-uniform wave velocity, two horizontal formation interfaces develop along the borehole axis (Figure 1): formation interface 1 (top) and formation interface 2 (bottom). In this case, the acoustic system is below formation interface 2, and the near-borehole cave is either inside (Figure 1a) or outside (Figure 1b) the interlayer. Upon encountering a formation interface, the borehole mode waves propagating upwards along the borehole axis are reflected and propagate downwards along the borehole axis. Meanwhile, upon entering the adjacent layered formation, the waves emitted by the acoustic source in the borehole are reflected and refracted at the formation interfaces. After multiple refractions at these interfaces and arrival at the near-borehole cave, any scattering point on the surface of the near-borehole cave acts as a secondary acoustic source, thus radiating scattered waves. After reaching a formation interface, these scattered echoes are again repeatedly reflected and refracted. Then, the receiver (R) records the scattered echoes after their refraction at the formation interfaces. Owing to the position of the logging instrument and axial heterogeneity in elastic-wave velocity in the near-borehole formation, the propagation paths of incident waves and scattered echoes differ for different scattering points on the surface of the near-borehole cave. Consequently, the wavefront of the intercepted scattered echoes from the near-borehole cave is no longer planar. Under these conditions, the arrival time of the scattered-echo head waves must be determined from the refraction and scattering paths of different elastic-wave modes.

Based on Fermat’s principle and Snell’s law of refraction, the two-point ray-tracing method for layered media can determine the complete acoustic-wave propagation path from the source to the receivers through the scattering point. Here, the positions of the transmitting and receiving transducers, measured longitudinal- and transverse-wave time differences of acoustic logging, and positions of the formation interfaces are known. The Newton–Raphson method is used to iteratively solve Equation (1) for determining the propagation path of the incident and scattered echoes in the layered formation exhibiting axial heterogeneity in elastic-wave velocities. The calculation formula is as follows:

(1) $$\left\{\begin{array}{c} f(p_{1})=p_{1}\sum_{s=1}^{\sigma }\frac{c^{T_{s}}H_{s}}{\sqrt{1-{\left(p_{1}c^{T_{s}}\right)}^{2}}}-\Delta_{0},\\ f(p_{2})=p_{2}\sum_{s=1}^{\sigma }\frac{c^{R_{s}}H_{s}}{\sqrt{1-{\left(p_{2}c^{R_{s}}\right)}^{2}}}-\Delta_{1},\\ p_{1}=\frac{\sin\theta_{s}}{c^{T_{s}}},p_{2}=\frac{\sin\theta_{s}}{c^{R_{s}}}, \end{array}\right.$$

where $\Delta_{0}$ denotes the radial distance between the acoustic source and scattering point, $\Delta_{1}$ denotes the radial distance between the scattering point and receiver, $c^{T_{s}}$ denotes the incident-wave velocity in the *s* layer, $c^{R_{s}}$ denotes the scattering-echo velocity in the *s* layer, $H_{s}$ denotes the thickness of the *s* layer, $\theta_{s}$ is the angle between the ray and normal parameters, σ denotes the number of layered strata, *p*_1_ denotes the ray parameter of the incident waves, *p*_2_ denotes the ray parameter of scattered echoes, $f(p_{1})$ denotes a nonlinear equation set corresponding to the incident waves, and $f(p_{2})$ denotes a nonlinear equation set corresponding to scattered echoes.

### 2.2. Three-Dimensional Acoustic Imaging for Detecting Anomalous Near-Borehole Bodies

The conventional downhole 3D spatial-scanning method for homogeneous formations assumes that elastic-wave propagation properties are spatially uniform in the near-borehole region. Under this assumption, the travel time of the scattered-echo head wave associated with a near-borehole scattering point is equal to the sum of the incident-wave travel time and the scattered-wave travel time. The start of the processing window for a waveform received at each receiving element of a cylindrical array can therefore be determined from the echo travel time. However, when formations with axially non-uniform wave velocities are present near a borehole, conventional imaging methods can produce significant errors in the reconstructed image of anomalous near-borehole geological bodies. The travel-time expression for the scattered-echo head wave associated with an arbitrary near-borehole scattering point in a homogeneous formation is given by

(2) $$\tau_{i,j}\left(x\right)=\frac{\left|x-x^{T}\right|}{c^{T_{1}}}+\frac{\left|x-x_{i,j}^{R}\right|}{c^{R_{1}}}$$

where *τ_i_*_,*j*_ is the first-arrival travel time of the scattered echo received by element *R_i_E_j_*, ***x*** is the spatial coordinate of a given grid node in the near-borehole formation, ***x****^T^* is the spatial coordinate of the transmitting transducer, and ***x****_i_*_,*j*_*^R^* is the spatial coordinate of *R*_i_*E*_j_.

The 3D spatial-scanning imaging of near-borehole anomalies in a formation with axially non-uniform wave velocities first requires the discretisation of the near-borehole formation into a grid. Assuming that each grid node represents a scattering point on the anomalous near-borehole body, the spatial relation between the node and the formation interface is established, and the corresponding formation wave-velocity parameters are determined. Considering an example where the acoustic system is below a single formation interface and a scattering point lies above this interface, the inversion procedure for borehole azimuthal acoustic imaging is derived as follows for a formation with axially non-uniform wave velocities. Notably, the slowness of the measured P- and S-waves in acoustic logging indicates variations in elastic-wave velocities along the borehole axis in the formation along with the position of the formation interface. Because the scattering point lies above the formation interface, the refraction of the incident waves and scattered echoes at the interface must be considered. The travel time of the scattered-echo head waves from any near-borehole scattering point is calculated as

(3) $$\left\{\begin{array}{l} \tau_{i,j}\left(x\right)=\frac{\left|x-x^{T}\right|}{c^{T_{1}}}+\frac{\left|x-x_{i,j}^{R}\right|}{c^{R_{1}}}(z\leq H),\\ \tau_{i,j}\left(x\right)=\frac{\left|x^{T}-\varepsilon^{T}\right|}{c^{T_{1}}}+\frac{\left|x-\varepsilon^{T}\right|}{c^{T_{2}}}+\frac{\left|x-\varepsilon_{i,j}^{R}\right|}{c^{R_{2}}}+\frac{\left|x_{i,j}^{R}-\varepsilon_{i,j}^{R}\right|}{c^{R_{1}}}(z>H), \end{array}\right.$$

where *τ_i_*_,*j*_ denotes the first-arrival travel time of scattered echoes corresponding to receiving elements *R_i_E_j_*, ***x*** denotes the spatial coordinate of any grid node in the near-borehole formation, ***x****^T^* denotes the spatial coordinate of the transmitting transducer, ***x****_i_*_,*j*_*^R^* denotes the spatial coordinate of receiving elements *R*_i_*E*_j_, ***ε****^T^* denotes the refraction point of the incident waves at the formation interface, and ***ε****_i_*_,*j*_*^R^* denotes the refraction point of the scattered echoes corresponding to receiving elements *R_i_E_j_* at the formation interface.

For the scattered echoes of a given mode, the positions of the transmitting and receiving transducers, position of the formation interface, and P- and S-wave velocities above and below the interface are known and used to calculate the travel time of the scattered-echo head waves for any near-borehole grid node. This travel time determines the window position in the corresponding received wave train. The echo waveform of the corresponding mode is extracted from each received wave train through windowing, and the similarity coefficient is calculated using Equation (4). Finally, all nodes in the near-borehole formation are traversed, and the node with the maximum similarity coefficient is identified as the actual scattering point [15].

(4) $$S\left(x\right)=\frac{\int_{0}^{T_{w}}{\left\{\sum_{i=1}^{N}\sum_{j=1}^{M}W_{i,j}\left[t+\tau_{i,j}\left(x\right)\right]\right\}}^{2}dt}{N\cdot M\cdot \int_{0}^{T_{w}}\sum_{i=1}^{N}\sum_{j=1}^{M}W_{i,j}^{2}\left[t+\tau_{i,j}\left(x\right)\right]dt},$$

where *S* denotes the similarity coefficient of the scattered echoes, *W*_i,j_ denotes the received waveforms corresponding to receiving elements *R_i_E_j_*, *N* denotes the number of acoustic receiving stations, *M* denotes the number of circumferential receiving elements in each receiving station, and *T*_w_ denotes the time-window length.

When determining echo propagation from of any near-borehole scattering point in formations with axially non-uniform wave velocities, the ray-tracing module used in this study uses a parallel computing framework. An iterative acceleration algorithm is incorporated to improve the path-solving process, which substantially reduces the computation time and increases the overall algorithmic efficiency. In this study, tests were conducted using an eight-core Intel i7-9700 processor with a main frequency of 3.00 GHz. For single-shot data, the 3D spatial-scanning imaging procedure required 2 min and consumed up to 20 GB of memory. These results show that the method can be executed without a high-performance computing cluster, underscoring its practicality and potential for broader application.

## 3. Characteristics of Scattered Echoes from Near-Borehole Caves

### 3.1. Numerical Model

**Figure 2:**
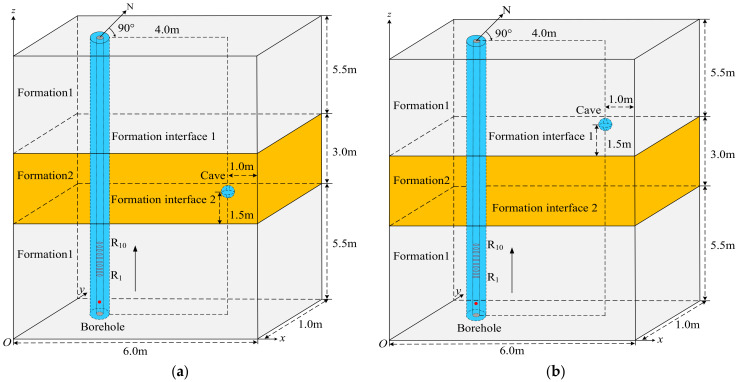
Schematic of the calculation model of near-borehole caves in a formation with axially non-uniform wave velocities: cave (**a**) within and (**b**) outside the interlayer.

The borehole azimuthal acoustic imaging response of near-borehole caves in a formation with axially non-uniform wave velocities was investigated. The finite-difference method was used in a 3D Cartesian coordinate system to simulate the borehole acoustic field of the near-borehole cave depicted in Figure 2 [24]. The model dimensions were 6.0 m × 1.0 m × 14.0 m. The borehole had a radius of 0.1 m. Its axis was aligned parallel to the z axis and located 0.5 m from the x axis and 1.0 m from the y axis. A cylinder composed of heavy mud with a radius of 0.03 m was used to simulate the instrument, and the axis of the acoustic system was aligned parallel to the borehole axis. The receiving array was a phased cylindrical acoustic array comprising 10 equally spaced stations, with a spacing of 0.2 m between adjacent stations and a minimum offset of 2.0 m. Formation interfaces 1 and 2 were located at z1 = 8.5 m and z2 = 5.5 m, respectively. The interlayer was designated as formation 2, and the formations above and below it were designated as formation 1. In the first and last measurements, the axial heights of the acoustic source were 1.0 and 10.0 m, respectively. The acoustic system was lifted nine times along the borehole axis in 1.0-m increments. The position of the acoustic source served as the reference depth when simulating multi-shot measurements during system lifting. A numerical model was also established for a formation with axially uniform wave velocities. The waveforms of scattered echoes from near-borehole caves in this model were compared with those in the case of the formation with axially uniform and non-uniform wave velocities. In this model, the interlayer was omitted, the formation adjacent to the borehole was designated as formation 1, and the cave location and other model parameters remained unchanged.

The cave was modelled as a fluid-filled sphere with a radius of 0.1 m (Figure 2). The radial distance between the cave centre and borehole axis was 4.0 m, and the axial heights were 7.0 and 10.0 m (Figure 2a and Figure 2b, respectively). The positive *y* direction was defined as north, and the clockwise direction was taken as positive. The cave azimuth angle was 90°. In the numerical simulation, the finite-difference grid spacing along the *x*, *y* and *z* directions was 0.005 m, the time step was 0.5 μs, the total simulation time was 8 ms, and the model contained 1200 × 200 × 2800 grids. Conventional perfectly matched layers (PMLs) lead to severe spurious reflections and even numerical instabilities under large grazing incidence angles, high wave impedance contrasts and anisotropic media [25,26,27]. Therefore, the convolutional PML was adopted because it exhibits superior absorption performance and improved computational stability. Table 1 lists the elastic parameters of each medium set in the calculation model.

**Figure 3:**
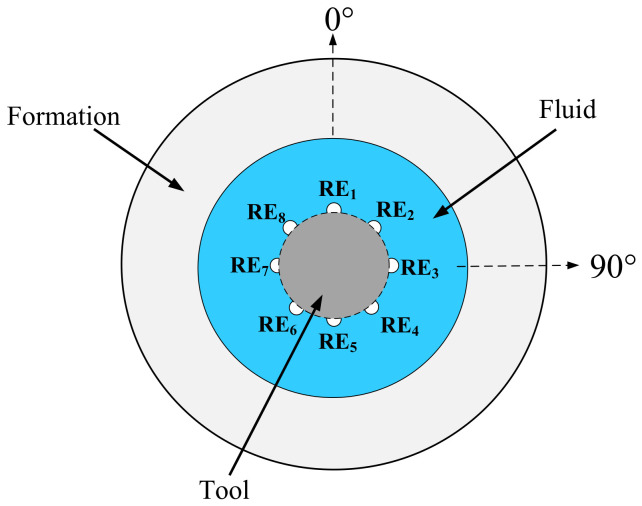
Top view of the simulation model.

From the inside outwards, the borehole comprised the instrument, a borehole fluid, and a layered formation with axially non-uniform wave velocities (Figure 3). Each acoustic receiving station comprised eight receiving elements (E_1_–E_8_) evenly distributed around the circumference. The azimuth angles ranged from 0° to 315°, with a circumferential interval of 45° between adjacent elements.

**Figure 4:**
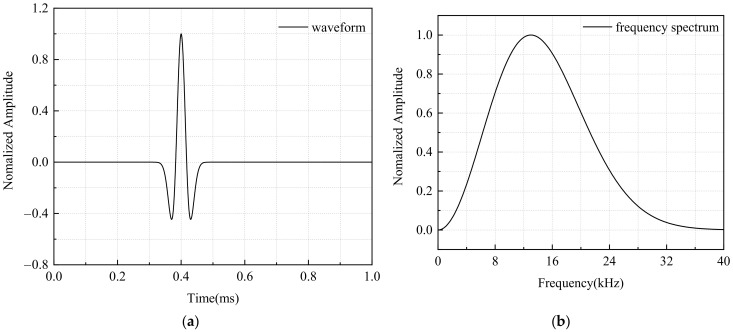
Normalised (**a**) time-domain waveform and (**b**) amplitude spectrum of the monopole acoustic source.

In the numerical simulation, the monopole acoustic source was simplified as a point source. Point source T was applied to each normal stress component, and a Ricker wavelet with a dominant frequency of 13 kHz was used as the source function. Figure 4 presents the normalised time-domain waveform and amplitude spectrum of the monopole acoustic source.

### 3.2. Waveform Feature Analysis

**Figure 5:**
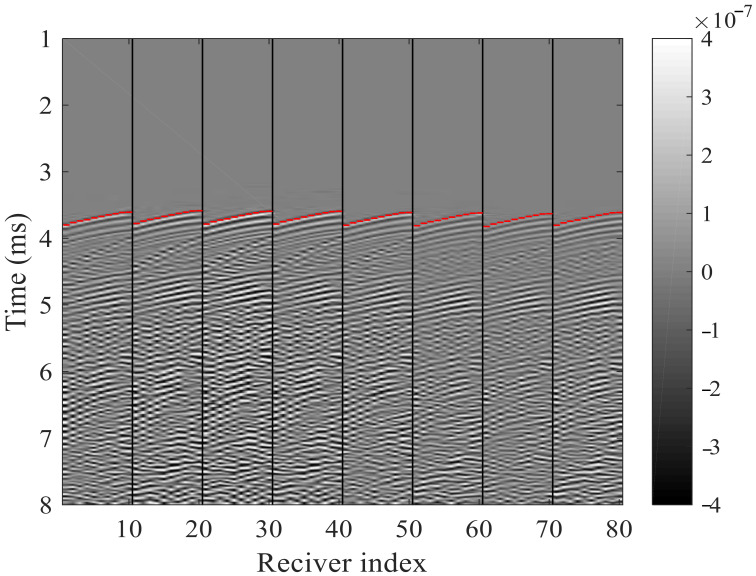
Variable-density map of scattered echoes generated by the near-borehole cave.

The small waveform amplitudes of the scattered echoes from the near-borehole cave made echo features difficult to identify in the full wave train. To isolate these features, the full waveforms computed using the cave-containing model were subtracted from those computed using the cave-free model. After eliminating interference from borehole mode waves and interface echoes, the variable-density map of the scattered echoes from the near-borehole cave located within the interlayer was obtained (Figure 5). The echoes were recorded using cylindrical array receivers during the first-shot measurement of the acoustic system. In Figure 5, the first channel from the left corresponds to the scattered echoes recorded by receiving elements R1E1–R10E1; the remaining channels follow the same order. In particular, the last channel corresponds to the scattered echoes recorded by receiving elements R1E8–R10E8, and the red dotted line marks the PP scattered echoes. The marked difference in the amplitudes of PP scattered echoes recorded by different azimuthal receiving elements indicate a clear azimuthal variation in the wave amplitude.

**Figure 6:**
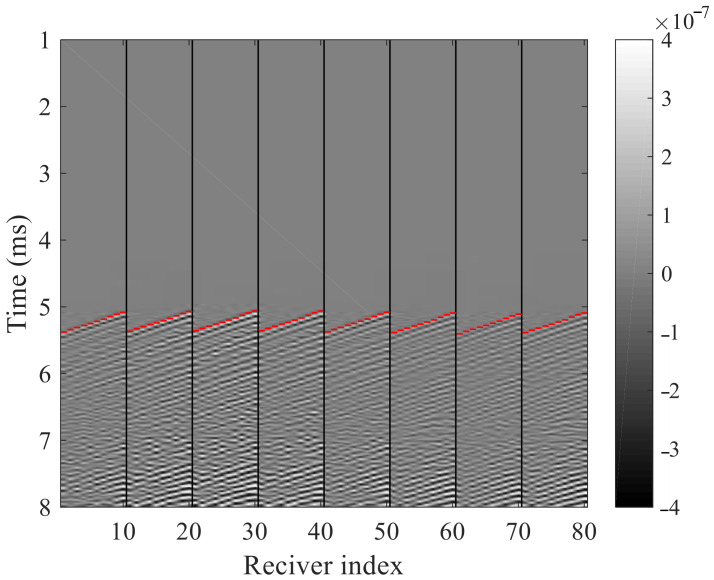
Variable-density map of scattered echoes from the near-borehole cave.

Figure 6 presents the variable-density plot of the scattered echoes from the near-borehole cave outside the interlayer recorded by the cylindrical array receivers during the first-shot measurement of the acoustic system. Notably, the gather naming convention in Figure 6 is the same as that in Figure 5, and the red dashed line marks the PP scattered echoes. The first arrival of the PP scattered echoes in Figure 6 is delayed compared with that in Figure 5.

**Figure 7:**
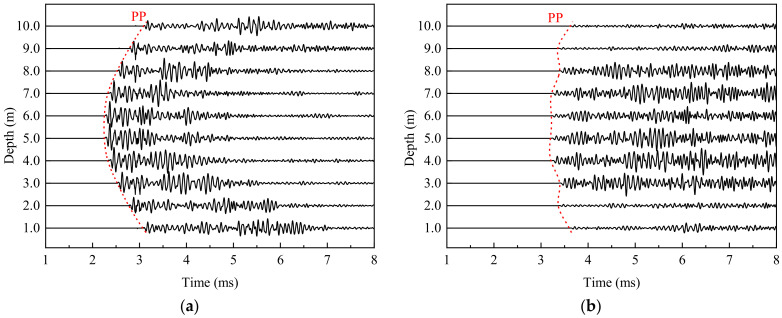
Scattered echoes from the near-borehole cave recorded by the receiving element R_6_E_3_ at a common offset of 3.0 m: (**a**) formation with axially uniform wave velocities and (**b**) formation with axially non-uniform wave velocities.

When the cave resides in the interlayer, Figure 7 shows the scattered echoes from the near-borehole cave recorded by the receiving element R_6_E_3_ under two conditions with a common offset of 3.0 m: a formation with axially uniform wave velocities with parameters consistent with those of formation 1 as well as a formation with axially non-uniform wave velocities. The red dotted line marks the PP scattered echoes. The vertical axis represents the axial height of the acoustic source, and all subgraphs use the same waveform scale.

When the acoustic system was located in the formation at a depth of 5.0–6.0 m, the amplitude of the scattered echoes from the near-borehole cave was the largest and the arrival time was the earliest (Figure 7a). As the acoustic system moved away from the deep cave end-point, the amplitude of the scattered echoes decreased gradually, and the arrival time was delayed. The in-phase axis showed a typical parabolic variation trend with respect to the depth.

In the formation with axially non-uniform wave velocities, the in-phase axis of the scattered echoes from the near-borehole cave varied asymmetrically with depth (Figure 7b). The echo amplitude was controlled by two factors: the axial distance between the acoustic system and near-borehole cave and the position of the acoustic system relative to the formation interface. Specifically, when the acoustic system moved within 1.0–2.0 and 9.0–10.0 m, the scattered echoes from the near-borehole cave showed small amplitudes. At these positions, the acoustic source and receiving element R_6_E_3_ were located outside—either above or below—the interlayer, whereas the cave remained inside the interlayer. Consequently, the incident waves and scattered echoes traversed the formation interface, which markedly reduced the echo amplitude. However, when the acoustic system moved within 3.0–8.0 m, the receiving element R_6_E_3_ and acoustic source successively entered the interlayer and lay in the same stratum as the cave. At this time, a part of the acoustic energy loss caused by refraction, reflection or scattering at the formation interface was suppressed, and the amplitude of the scattered echoes increased overall.

## 4. Three-Dimensional Single-Shot Imaging of Scattered Echoes

### 4.1. Cave Located Within the Interlayer

**Figure 8:**
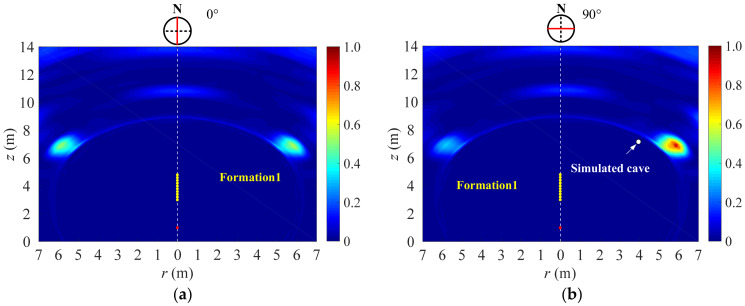
Similarity images based on PP scattered echoes in a formation with uniform wave velocities at different azimuths crossing the borehole axis: (**a**) 0° azimuth and (**b**) 90° azimuth.

Regardless of the presence of the interlayer, the formation adjacent to the borehole was assumed to be formation 1. Furthermore, traditional downhole 3D spatial-scanning imaging for homogeneous formations was employed to generate the similarity images of PP scattered echoes (Figure 8) based on data obtained from the near-borehole cave shown in Figure 5 in two borehole cross-sections: one perpendicular to the sagittal plane of the simulated cave-scattered waves at 0° azimuth and the other parallel to it at 90° azimuth. In Figure 8, the red dots represent the acoustic source, yellow dots represent the acoustic receiving stations, and white dashed line represents the borehole axis. The similarity coefficient of the PP echoes was largest at the 90° azimuth, but the position of the similarity maximum (red star in Figure 8) still differed markedly from the centre of the simulated cave (white circle). The radial-distance error between the two was approximately 1.8 m, and the axial-height error was approximately 0.2 m.

**Figure 9:**
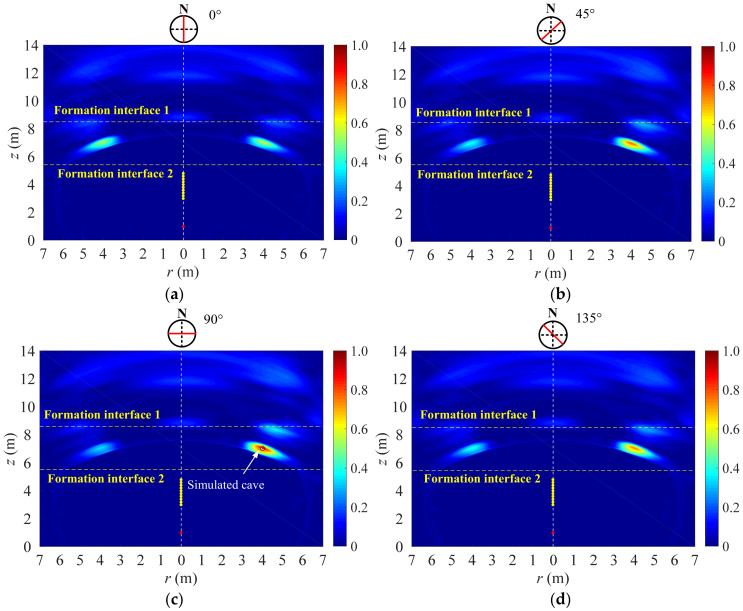
Similarity images based on the PP scattered echoes in profiles crossing the borehole axis at different azimuths in the formation with axially non-uniform wave velocities: (**a**) 0° azimuth; (**b**) 45° azimuth; (**c**) 90° azimuth; (**d**) 135° azimuth. (The cave located within the interlayer).

Next, the proposed method was used to generate the similarity images of PP scattered echoes (Figure 9) based on the data from the near-borehole cave shown in Figure 5. The images were generated in profiles crossing the borehole axis at different azimuths in the formation with axially non-uniform wave velocities (Figure 9). The yellow dashed lines in Figure 9 denote the formation interfaces, with the upper and lower interfaces labelled as formation interfaces 1 and 2, respectively. Because the acoustic system was located below formation interface 2 during the first-shot measurement, refraction of the incident waves and scattered echoes at formation interfaces 1 and 2 had to be considered when spatially scanning any grid node above formation interface 1. In the spatial scanning of any grid node within the interlayer, refraction of the incident waves and scattered echoes at formation interface 2 had to be considered. By contrast, the refraction of acoustic waves at the formation interfaces could be ignored when spatially scanning any grid node below formation interface 2. Overall, the similarity coefficient of the PP scattered echoes was largest in the 90-azimuth imaging profile passing through the borehole axis, and the simulated cave was imaged most clearly in that profile (Figure 9).

The maximum similarity coefficient of the PP scattered echoes in Figure 9c was located at (4.04 m, 7.02 m). Given that the radial distance between the centre of the simulated cave and borehole axis was 4.0 m and the axial height was 7.0 m, the radial and axial positioning errors were 0.04 and 0.02 m, respectively. Overall, the maximum similarity of the PP scattered echoes (red star in Figure 9) closely aligned with the centre of the simulated cave (white circle). The 3D spatial coordinates of the cave were determined based on these positioning results. In the first-shot measurement of the acoustic system, the actual azimuth and vertical angles of the centre of the near-borehole cave relative to the centre of the cylindrical array acoustic receiving station were 90° and 37.8°, respectively. Correspondingly, the errors in the azimuth and vertical angles of the simulated cave measured by the proposed method were 0° and 0.1°, respectively.

When the cave was located within the interlayer, the spatial coordinates associated with the peak similarity coefficients of the PP scattered echoes in Figure 8b and Figure 9c were extracted. These coordinates were then compared with the true positions of the simulated cave to evaluate the positioning error, with the results summarised in Table 2. The conventional imaging method yielded larger positioning errors. By contrast, the proposed method, which combines ray tracing with spatial scanning, yielded much smaller positioning errors and substantially improved imaging accuracy.

### 4.2. Cave Located Outside the Interlayer

**Figure 10:**
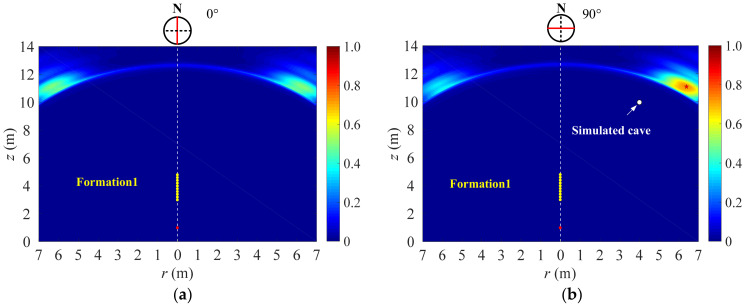
Similarity images based on the PP scattered echoes in a section with the azimuth crossing the borehole axis in a formation with axially uniform wave velocities: (**a**) 0° azimuth; (**b**) 90° azimuth.

Regardless of the presence of the intermediate interlayer, formation 1 was assumed to be present, adjacent to the borehole. Traditional downhole 3D spatial-scanning imaging for homogeneous formations was used to generate the similarity images of PP scattered echoes (Figure 10) based on the data from the near-borehole cave shown in Figure 6. The images were generated in two sections crossing the borehole axis: one perpendicular to and the other parallel to the sagittal plane of the scattered waves from the simulated cave. The spatial error between the position of the similarity-coefficient maximum (red star in Figure 10) and the centre of the simulated cave (white circle) was substantial. The radial-distance error between the two was approximately 2 m, and the axial-height error was approximately 1 m.

**Figure 11:**
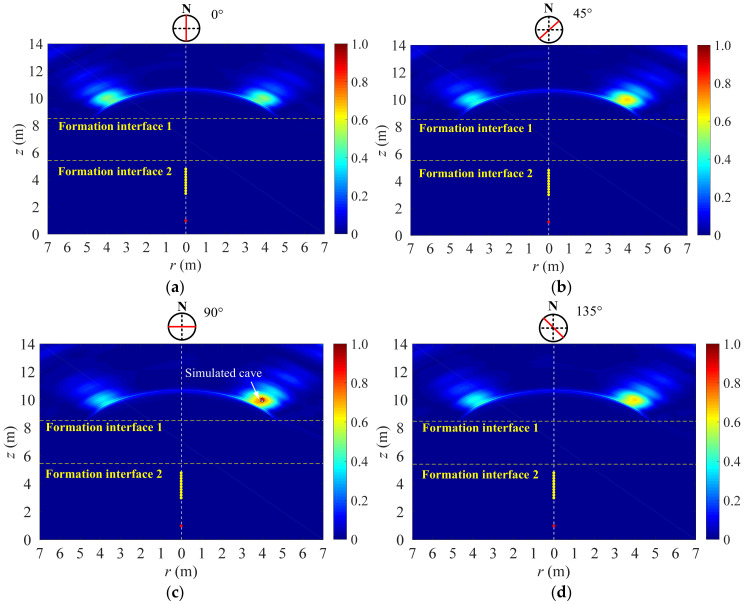
Similarity images based on PP scattered echoes in profiles crossing the borehole axis at different azimuths in the formation with axially non-uniform wave velocities: (**a**) 0° azimuth; (**b**) 45° azimuth; (**c**) 90° azimuth; (**d**) 135° azimuth (The cave located outside the interlayer).

Next, the proposed method was used to generate the similarity images of PP scattered echoes (Figure 11) based on the data from the near-borehole cave shown in Figure 6 in profiles crossing the borehole axis at different azimuths in the formation with axially non-uniform wave velocities. The similarity coefficient of the PP scattered echoes was largest in the 90-azimuth imaging section passing through the borehole axis, and the simulated cave was imaged most clearly in that section (Figure 11). The maximum similarity coefficient (red star in Figure 11) closely aligned with the centre of the simulated cave (white circle).

The maximum similarity coefficient of the PP scattered echoes in Figure 11c was located at (4.02 m, 9.98 m). Given that the radial distance between the cave centre and borehole axis was 4.0 m and the axial height was 10.0 m, the radial and axial positioning errors were both 0.02 m. The 3D spatial coordinates of the cave were then determined based on these positioning results. In the first-shot measurement of the acoustic system, the true azimuth and vertical angles of the cave centre relative to the centre of the cylindrical array acoustic receiving station were 90° and 56.7°, respectively. Accordingly, errors in the azimuth and vertical angles of the simulated cave measured by the proposed method were 0° and 1.2°, respectively.

When the cave was located outside the interlayer, the spatial coordinates corresponding to the peak similarity coefficients of the PP scattered echoes in Figure 10b and Figure 11c were extracted. Then, they were compared with the true coordinates of the simulated cave to evaluate the positioning error. The corresponding results are summarised in Table 3. The conventional imaging method exhibited a large positioning error, whereas the proposed method achieved markedly higher positioning accuracy.

### 4.3. Influence of Noise on Imaging Results

To evaluate how noise affects the imaging results of simulated near-borehole caves in formations with axially non-uniform wave velocities, Gaussian white noise was added to the scattered-echo waveforms shown in Figure 5 and Figure 6 and directly superimposed on the time-domain array signals. After noise contamination, the time-domain signal-to-noise ratio (SNR) is expressed as

(5) $$\mathrm{SNR}=10\log_{10}\frac{A}{A_{Noise}}$$

where *A* is the power of scattered-echo signals and *A_Noise_* is the power of the Gaussian white noise. The SNR is expressed in decibels (dB).

**Figure 12:**
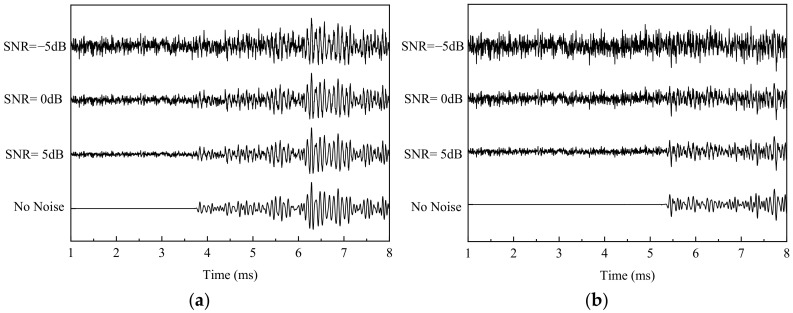
Scattered echoes from a near-borehole cave before and after noise addition: cave (**a**) within and (**b**) outside the interlayer.

Figure 12 shows the echo waveforms recorded by *R*_1_*E*_1_ under noise-free conditions and at SNRs of −5, 0 and 5 dB when the cave was located inside (Figure 12a) and outside the interlayer (Figure 12b). The PP echo characteristics remained distinct at SNRs of 0 and 5 dB. However, when the SNR decreased to −5 dB, the waveforms were strongly contaminated by noise, and no clearly identifiable PP echo features remained.

**Figure 13:**
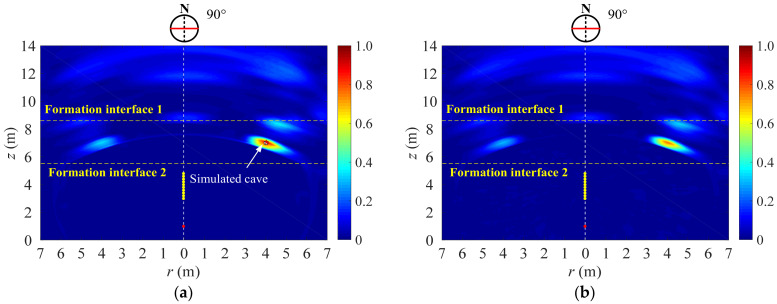

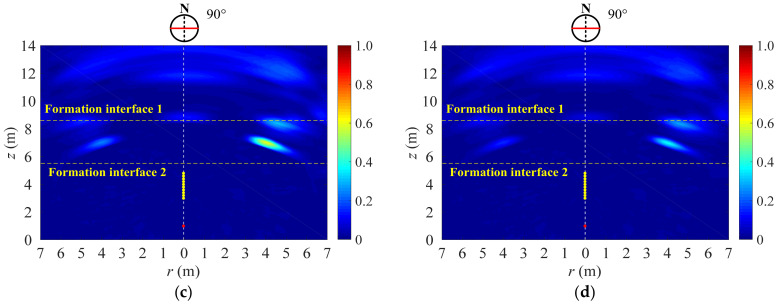
Similarity results based on PP scattered echoes in a 90-azimuth (cross-borehole) profile for a cave adjacent to a borehole within an interlayer obtained under different SNR conditions: (**a**) no noise; (**b**) SNR = 5 dB; (**c**) SNR = 0 dB; and (**d**) SNR = −5 dB.

Figure 13 presents the PP scattered-wave similarity results in a 90° azimuth (cross-borehole) profile obtained using the proposed method for a cave adjacent to a well within an interlayer under noise-free and noisy conditions. As the SNR of the echo signals gradually decreased, the peak PP-wave similarity coefficient decreased along with the intensity of the imaging energy cluster.

**Figure 14:**
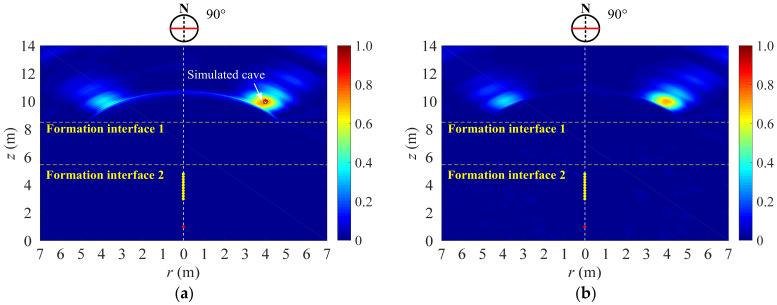

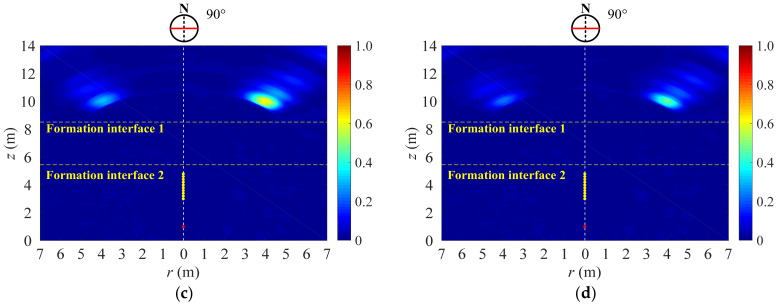
Similarity imaging results based on PP scattered echoes in the 90-azimuth (cross-borehole) profile for a cave located above the interlayer obtained under different SNR conditions: (**a**) no noise; (**b**) SNR = 5 dB; (**c**) SNR = 0 dB; and (**d**) SNR = −5 dB.

Figure 14 shows the corresponding PP scattered-echo similarity results in the 90-azimuth profile for a cave located above the interlayer obtained using the proposed method with and without noise contamination.

In Figure 13 and Figure 14, the spatial coordinates corresponding to the peak PP-wave similarity coefficients remain clearly identifiable at SNRs of 0 and 5 dB. This indicates that the proposed imaging method is robust against noise. This behaviour can be attributed to the fact that random noise has substantially lower similarity across the multichannel received waveforms compared with target echo signals. However, at an SNR of −5 dB, the noise energy greatly exceeded the energy of the target signal, and the target echoes were fully contaminated by noise. Consequently, the PP-wave similarity coefficients in the imaging map decreased, the imaging SNR decreased, and the imaging quality of the simulated cave became noticeably poor.

## 5. Multi-Shot Stack Imaging

**Figure 15:**
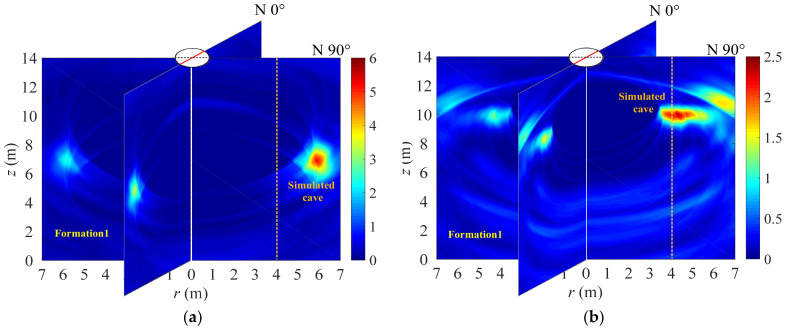
Multi-shot stacked scanning images based on PP scattered-echo data in the formation with axially uniform wave velocities at different azimuths crossing the borehole axis: (**a**) cave within the interlayer and (**b**) cave above the interlayer.

**Figure 16:**
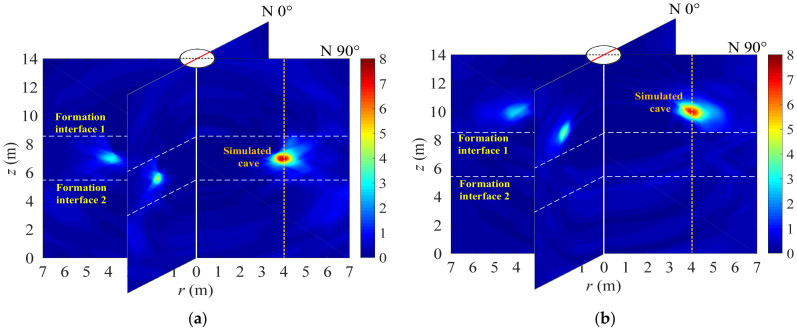
Multi-shot stacked scanning images based on PP scattered-echo data in the formation with axially non-uniform wave velocities at different azimuths crossing the borehole axis: (**a**) cave within the interlayer and (**b**) cave above the interlayer.

The scale, orientation and depth of the simulated cave were estimated by superimposing the single-shot data from all depth points on the vertical-plane imaging map. Images from two profiles crossing the borehole axis—one parallel to and the other perpendicular to the azimuthal plane of the scattered waves from the simulated cave—were selected to obtain clear imaging results for analysis. Figure 15 presents the multi-shot stacked images produced by traditional downhole 3D spatial-scanning imaging for uniform formations. These results were obtained from the PP scattered-echo data of near-borehole caves in formations with axially non-uniform wave velocities, with the formation adjacent to the borehole assumed to be formation 1. Figure 16 presents the corresponding multi-shot stacked imaging results based on PP scattered echoes obtained by the proposed method for near-borehole caves located inside and outside the interlayer. The yellow dashed lines in Figure 15 and Figure 16 mark the true radial position of the simulated cave.

Notably, traditional downhole 3D spatial scanning assumes uniform axial propagation of elastic waves in near-borehole formations. Therefore, in the presence of a near-borehole formation with axially non-uniform wave velocities, the position of the maximum similarity coefficient of scattered echoes differed across shot measurements of the acoustic system and could not accurately reflect the true position of the anomalous near-borehole body. Additionally, after multi-shot stacked imaging, the imaging error of the anomalous near-borehole body increased further. Specifically, the radial-distance error of the simulated cave in Figure 15a reached approximately 2 m, and several similarity-coefficient maxima appeared in Figure 15b, with the maximum point deviating from the true position of the simulated cave.

By contrast, the proposed method for borehole azimuthal acoustic imaging based on ray tracing and spatial scanning fully accounted for the propagation paths of multiple refracted incident waves and scattered echoes in formations with axially non-uniform wave velocities. Consequently, it achieved high-precision imaging of near-borehole caves under such formation conditions. As illustrated in Figure 16, the radial-distance and depth errors of the simulated cave were small. Furthermore, the comparison of the single-shot spatial-scanning imaging results for the near-borehole cave in Figure 9 and Figure 11 indicated that after multi-shot stacked imaging, the cave contour became clearer, azimuthal distribution characteristics became more distinct, and artefact interference was substantially reduced.

The spatial coordinates corresponding to the maximum similarity coefficients of the PP scattered echoes in the multi-shot stacked imaging results were extracted for two cases: when the karst cave was located inside the interlayer and when it was located above the interlayer. These coordinates were then compared with the true positions of the simulated karst caves to evaluate the positioning error (Table 4). The positioning accuracy of the conventional downhole 3D spatial-scanning imaging method was considerably affected by formation heterogeneity pertaining to wave velocity in the axial direction. When the karst cave was located inside the interlayer, the radial positioning error reached 1.92 m. Even when the karst cave was located above the interlayer—so that several final measurement shots of the acoustic tool were recorded in the same formation as the cave—the radial error reached 0.52 m. By contrast, the proposed method achieved high positioning accuracy under both scenarios.

## 6. Discussion

This section presents an in-depth analysis and discussion of the imaging results of the simulated caves described above. The performances of the proposed method and the conventional method are compared in terms of positioning accuracy and noise robustness, with the aim of clarifying the technical advantages and underlying mechanism of the proposed method for acoustic imaging of anomalous near-borehole bodies in formations with axially non-uniform wave velocities.

Taking the cave above the interlayer as an example, the true radial distance from the cave centre to the borehole axis is 4 m, and its axial height is 10 m. As shown in Table 3, the two imaging methods differ substantially in their positioning accuracy for the simulated cave. For the conventional method, the absolute error in radial distance reaches 2.38 m, corresponding to a relative error of 59.5%, whereas the absolute error in axial height reaches 1.06 m, corresponding to a relative error of 10.6%. This level of imaging accuracy is clearly insufficient for practical field applications. This is because the conventional method uses the first-arrival travel-time expression for scattered echoes from arbitrary near-borehole scattering points in a homogeneous formation, as given in Equation (2). It neglects the effects of formation interfaces and interface-induced refraction, leading to large errors in the calculated echo travel times and, ultimately, to spatial positioning shifts and blurred imaging contours of anomalous near-borehole geological bodies.

By contrast, the proposed azimuthal acoustic remote-detection imaging method, which combines ray tracing with spatial scanning, produces absolute errors of only 0.02 m in both the radial distance and axial height of the simulated cave, with relative errors of 0.5% and 0.2%, respectively. The positioning accuracy of the simulated cave is therefore greatly improved, and the errors are within an acceptable range for engineering applications, indicating good potential for field implementation. Because the proposed method is based on ray tracing, it can accurately calculate the propagation paths of the scattered echoes associated with arbitrary near-borehole scattering points in formations with axially non-uniform wave velocities and obtain accurate first-arrival travel times of the scattered echoes. It therefore avoids, at the source, the imaging errors caused by the homogeneous-velocity assumption used in conventional imaging methods. The multi-shot stacked imaging results obtained using different methods further support this conclusion and are therefore not repeated here.

Comparison of the imaging results of the simulated caves under different noise conditions in Figure 13 and Figure 14 shows that the similarity coefficient of the PP scattered echoes gradually decreases as the signal-to-noise ratio (SNR) of the echo signals decreases. Specifically, compared with the noise-free case, the spatial positions corresponding to the maximum similarity coefficient of the PP echoes remain clearly identifiable at both SNR = 5 dB and SNR = 0 dB, indicating that the proposed method has good noise robustness. This method performs imaging based on waveform similarity and makes comprehensive use of the arrival time, amplitude, phase and wavelet duration of the echo signals. The receiving elements in the array record scattered echoes of the same mode that are generated by the same downhole acoustic source and scattered from the same near-borehole scattering point; these effective echo signals therefore exhibit strong waveform similarity. By contrast, random noise has no fixed coherent characteristics and exhibits extremely low waveform similarity. As a result, the proposed method can effectively suppress interference from common random noise. However, when the SNR decreases to −5 dB, the noise energy is much higher than the energy of the effective signals, and the useful echo signals are buried by strong noise. Consequently, the similarity coefficient of the PP scattered echoes in the imaging results decreases markedly, and the imaging features of the simulated cave become weakened. It should be noted that such extreme noise-contamination conditions are uncommon in field acoustic logging and are usually associated with abnormal downhole tool operation.

In summary, the proposed imaging method can meet the application requirements of acoustic remote detection logging technology in the exploration and development of heterogeneous reservoirs and structurally complex reservoirs, and it therefore has practical engineering value. Nevertheless, the method still has certain limitations and applicable conditions, which are further discussed in Section 7.

## 7. Conclusions

This article proposes an acoustic remote detection imaging method combining ray tracing and spatial scanning. Scattered-echo data from near-borehole caves in formations with axially non-uniform velocities were obtained through numerical forward modelling. Further, the imaging results of a simulated cave obtained using the traditional imaging method (which assumes homogeneous formations) were compared with those obtained using the proposed method. The following results were obtained.

In formations with axially uniform wave velocities, as the acoustic logging tool moved away from the cave depth, the amplitude of the scattered echoes from the near-borehole cave decreased and the arrival time was increased, with the waveform exhibiting a typical parabolic shape as a function of depth. Conversely, in formations with axially non-uniform wave velocities, regardless of whether the near-borehole cave was inside or outside the interlayer, the scattered-echo waveforms in the common-offset waveform profile exhibited an asymmetric, depth-dependent variation pattern. The echo amplitude was jointly controlled by the axial distance between the acoustic tool and cave as well as the position of the tool relative to the formation interface.

Compared with conventional imaging, the proposed method for borehole azimuthal acoustic imaging incorporating ray tracing and spatial scanning enabled the accurate imaging of near-borehole anomalies in formations with axially non-uniform wave velocities and the precise estimation of the radial distance, azimuth and depth of simulated caves. Single-shot spatial-scanning imaging and multi-shot stack imaging results verified the effectiveness and reliability of the proposed method. Further related field tests will be prioritised in future research.

This study adopted a representative horizontally layered formation model in acoustic logging to validate the proposed method. In practice, formation interfaces feature undulations and are not perfectly planar. Because downhole measurements are restricted, obtaining detailed information on interface geometry is difficult; therefore, logging applications often treat formation interfaces as horizontal for simplicity. This simplifying assumption inevitably introduces errors in echo travel-time estimation and, to some extent, reduces the positioning accuracy of near-well anomalous bodies. Moreover, the present study does not account for several field-related perturbations. Under high-temperature and high-pressure downhole conditions, unstable instrument performance can lower the SNR of the echo signals, and borehole enlargement and mud invasion may further attenuate acoustic waves. Consequently, the field performance and practical engineering applicability of the proposed method still require further in-depth investigation in future.

## Figures and Tables

**Table 1:** Parameters used in the numerical simulation.

Medium	*V_p_* (m/s)	*V_s_* (m/s)	*ρ* (kg/m^3^)
Fluid	1500	—	1000
Formation 1	4500	2600	2500
Formation 2	2700	1600	2000

**Table 2:** Positioning results of the near-borehole cave obtained by different imaging methods (The cave located within the interlayer).

Imaging Method	Parameter	True Value (m)	Inverted Value (m)	Absolute Error (m)
Conventional method	Radial distance	4.0	5.88	1.88
	Axial height	7.0	6.86	0.14
Proposed method	Radial distance	4.0	4.04	0.04
	Axial height	7.0	7.02	0.02

**Table 3:** Positioning results of the near-borehole cave obtained by different imaging methods (The cave located outside the interlayer).

Imaging Method	Parameter	True Value (m)	Inverted Value (m)	Absolute Error (m)
Conventional method	Radial distance	4.0	6.38	2.38
	Axial height	10.0	11.06	1.06
Proposed method	Radial distance	4.0	4.02	0.02
	Axial height	10.0	9.98	0.02

**Table 4:** Comparison of positioning errors in the multi-shot stacked imaging of the cave adjacent to the borehole.

Cave Position	Imaging Method	Measured Parameter	True Value (m)	Inverted Value (m)	Absolute Error (m)
Inside the interlayer	Conventional method	Radial distance	4.0	5.92	1.92
		Axial height	7.0	6.88	0.12
	Proposed method	Radial distance	4.0	4.02	0.02
		Axial height	7.0	6.98	0.02
Above the interlayer	Conventional method	Radial distance	4.0	4.52	0.52
		Axial height	10.0	9.92	0.08
	Proposed method	Radial distance	4.0	4.0	0
		Axial height	10.0	9.98	0.02

## Data Availability

The original contributions presented in this study are included in the article. Further inquiries can be directed to the corresponding author.

## Abbreviations

The following abbreviations are used in this manuscript:

PP	Incident P-waves scattered as P-waves
PML	Perfectly matched layer
SNR	Signal-to-noise ratio
